# Temporal Dynamics and Clinical Relevance of Oscillatory Amplitude Variability During High‐Frequency Oscillatory Ventilation With Volume Guarantee in Preterm Infants

**DOI:** 10.1002/ppul.71859

**Published:** 2026-10-04

**Authors:** Kamal Ali, Mesaed Alsenani, Saad Alshareedah, Mohamed Sufyani, Saleh Algarni, Piero Alberti, Abdulaziz Homedi, Saif Alsaif, Ibrahim Ali, Theodore Dassios, Anne Greenough

**Affiliations:** ^1^ Neonatal Intensive Care Department King Abdulaziz Medical City Riyadh Saudi Arabia; ^2^ King Abdullah International Medical Research Centre Riyadh Saudi Arabia; ^3^ College of Medicine King Saud Bin Abdulaziz University for Health Sciences Riyadh Saudi Arabia; ^4^ Neonatal Intensive Care Centre King's College Hospital London UK; ^5^ Department of Women and Children's Health School of Life Course Sciences, Faculty of Life Sciences and Medicine King's College London London UK

## Abstract

**Objective:**

To characterise temporal dynamics, variance structure, determinants, and predictive relevance of oscillatory amplitude variability during high‐frequency oscillatory ventilation with volume guarantee (HFOV‐VG) in preterm infants.

**Methods:**

Single‐centre retrospective cohort study using minute‐level ventilator data collected during the first 72 h of HFOV‐VG. Amplitude variability was quantified using standard deviation, coefficient of variation (CV), and root mean square of successive differences (RMSSD). Temporal patterns, variance decomposition, and determinants of variability were assessed using mixed‐effects modelling separating within‐ and between‐infant effects. Predictive performance for death before discharge or moderate‐to‐severe bronchopulmonary dysplasia was evaluated using receiver operating characteristic analysis.

**Results:**

A total of 237,790 measurements from 60 infants were analysed. Amplitude CV declined from 0.122 (0.072–0.160) at 0–6 h to 0.063 (0.035–0.106) at 48–72 h, while RMSSD decreased from 2.10 (1.22–2.69) to 1.13 (0.64–1.94). Variability was predominantly within‐infant (ICC 0.246–0.282), whereas mean amplitude, VThf/kg, and frequency were predominantly between‐infant. Within‐infant increases in oscillatory frequency were associated with higher variability, whereas increases in mean airway pressure and time from HFOV initiation were associated with lower variability. The composite outcome occurred in 47/60 infants. Early oscillatory amplitude variability demonstrated moderate discrimination (AUC 0.72, 95% CI 0.55–0.86), with an AUC of 0.82 (95% CI 0.66–0.95) when combined with oscillatory frequency and weight‐normalised DCO_2_.

**Conclusions:**

Oscillatory amplitude variability during HFOV‐VG reflects dynamic ventilator–lung interactions. Its association with adverse respiratory outcome is hypothesis‐generating and requires validation in larger independent cohorts.

## 1 Introduction

High‐frequency oscillatory ventilation (HFOV) is an established mode of respiratory support in preterm infants with respiratory failure, particularly in the setting of evolving lung disease and heterogeneous pulmonary mechanics. Gas exchange during HFOV depends on oscillatory pressure transmission and is influenced by oscillatory amplitude, frequency, mean airway pressure, and underlying lung mechanics [1, 2].

The addition of volume guarantee (VG) during HFOV aims to stabilise tidal volume delivery through automatic adjustment of oscillatory amplitude in response to changes in respiratory mechanics [3]. Recent evidence, including a systematic review of HFOV‐VG in infants, has further characterised the physiological and clinical effects of this ventilation strategy [4]. HFOV‐VG has been associated with more consistent delivery of oscillatory tidal volume and improved control of carbon dioxide elimination compared with conventional HFOV strategies [5, 6, 7]. Nevertheless, oscillatory amplitude frequently varies during clinical use, and the physiological significance of this variability remains incompletely understood.

Previous studies have focused predominantly on relationships between oscillatory tidal volume, oscillatory frequency, diffusion coefficient of carbon dioxide (DCO_2_), and carbon dioxide elimination during HFOV [8, 9, 10, 11]. In contrast, the temporal behaviour of oscillatory amplitude itself has received limited attention. It remains unclear whether variability in oscillatory amplitude reflects evolving ventilator–lung interaction and dynamic changes in respiratory mechanics or represents non‐specific ventilator adjustment.

Analysis of high‐resolution time‐series data may provide additional insight into these dynamic physiological processes by allowing separation of within‐infant temporal variability from between‐infant differences in disease severity and ventilatory characteristics. Such an approach may help characterise the physiological significance of amplitude variability over time.

The aim of this study was to characterise the temporal evolution and variance structure of oscillatory amplitude during HFOV‐VG and to examine its physiological determinants using time‐resolved modelling. We further sought to distinguish within‐infant dynamic effects from between‐infant variability and to evaluate whether early oscillatory amplitude variability provides clinically relevant information beyond conventional ventilator‐derived parameters, including exploratory assessment of discrimination for death or moderate‐to‐severe bronchopulmonary dysplasia.

## 2 Methods

### 2.1 Study Design and Setting

This retrospective cohort study was conducted in the tertiary neonatal intensive care unit at King Abdulaziz Medical City (KAMC), Riyadh, Saudi Arabia, between November 2024 and February 2026. The unit provides level III neonatal care and uses high‐frequency oscillatory ventilation with volume guarantee (HFOV‐VG) as part of respiratory management for preterm infants when clinically indicated. All infants were ventilated using the Dräger VN600 ventilator platform (Dräger Medical, Lübeck, Germany).

### 2.2 Study Population

Preterm infants born at less than 30 weeks of gestation who received HFOV‐VG during the study period were eligible. Infants were identified from the unit's clinical database and ventilator data recording systems, and demographic and baseline clinical characteristics were obtained from electronic medical records. During the study period, 87 potentially eligible infants were identified; 25 did not receive HFOV‐VG, and two of the 62 infants who received HFOV‐VG were excluded because of underlying genetic abnormalities, resulting in a final analytical cohort of 60 infants. All eligible infants meeting the prespecified inclusion criteria during the study period were included; therefore, the sample size was determined by the available retrospective cohort, and no prospective sample‐size calculation was performed.

This cohort overlaps with that included in our previously published analysis of ventilatory efficiency during HFOV‐VG [12]. The previous study examined determinants of carbon dioxide clearance and ventilatory efficiency, whereas the present study addresses a distinct research question focused on the temporal dynamics, variance structure, physiological determinants, and prognostic relevance of oscillatory amplitude variability.

### 2.3 Inclusion and Exclusion Criteria

Infants were included if minute‐level ventilator data were available and sufficient to derive the prespecified variability measures during the first 72 h after NICU admission/initiation of HFOV‐VG. All included infants commenced HFOV‐VG on NICU admission, which was defined as time zero for longitudinal analyses. Infants were excluded if ventilator recordings were insufficient for analysis, key analytical variables were missing, or data quality was inadequate to derive time‐resolved variability measures.

### 2.4 HFOV‐VG Management

Typical initial HFOV‐VG settings included a target VThf of ~1.5–1.9 mL/kg, oscillatory frequency of 15 Hz (adjusted within ~15–17 Hz when clinically indicated), and MAP of approximately 10 mbar. Lung recruitment was performed by stepwise MAP adjustment to optimise oxygenation and lung expansion while avoiding overdistension, followed by titration to the lowest MAP maintaining adequate recruitment. An I:E ratio of 1:2 or 1:1 was used according to clinical circumstances. Opioid analgesia/sedation was used in all infants during HFOV‐VG; neuromuscular blocking agents were not used. Subsequent adjustments in VThf, frequency, MAP, and FiO_2_ were guided by oxygenation, carbon dioxide clearance, delivered VThf, blood gases, and clinical assessment rather than by a fixed gestational‐age‐ or weight‐based algorithm.

### 2.5 Data Acquisition

Ventilator data were extracted directly from the ventilator system at minute‐level frequency during the first 72 h after NICU admission/initiation of HFOV‐VG. Recorded variables included oscillatory amplitude, measured tidal volume (VThf), oscillatory frequency, mean airway pressure (MAP), fraction of inspired oxygen (FiO_2_), and diffusion coefficient of carbon dioxide (DCO_2_). DCO_2_ was calculated as VThf^2^ × oscillatory frequency (mL^2^/s) and normalised to body weight (DCO_2_/kg; mL^2^/s/kg).

Data were aligned to NICU admission/initiation of HFOV‐VG (time zero) and analysed across predefined epochs of 0–6, 6–12, 12–24, 24–48, and 48–72 h. Infants contributed to each epoch when valid electronically captured data were available. Although all infants commenced HFOV‐VG on NICU admission, electronic recording did not begin uniformly from admission in every infant; consequently, infants whose recordings commenced later did not contribute to earlier epochs, accounting for the smaller numbers in the 0–6 and 6–12 h epochs.

### 2.6 Data Preprocessing and Derivation of Variability Metrics

VThf and DCO_2_ were normalised to birth weight. The ventilator export provided one recorded value for each variable at each available minute‐level timestamp, which was used without additional averaging before hourly aggregation. Minute‐level observations were grouped into non‐overlapping hourly intervals. Within each infant‐hour, mean oscillatory amplitude and standard deviation (SD) were calculated, and the coefficient of variation (CV) was defined as SD divided by mean amplitude.

RMSSD was calculated as the square root of the mean squared difference between successive amplitude measurements separated by exactly 1 min. Pairs spanning missing timestamps were excluded, and missing observations were not imputed.

No automatic numerical exclusion was applied solely on the basis of extreme amplitude values. The ventilator export did not contain reliable event flags allowing observations to be attributed to transient events such as suctioning, circuit disconnection, accidental extubation, or flow‐sensor malfunction; therefore, stable amplitude alone was not used as an exclusion criterion. As no minimum number of minute‐level observations per hourly window had been prespecified, a sensitivity analysis requiring at least 30 valid amplitude observations per hour was performed to assess the robustness of the hourly variability estimates.

### 2.7 Outcomes

The primary physiological outcomes were oscillatory amplitude variability measured at the hourly level using CV and RMSSD. Secondary physiological measures included oscillatory amplitude, weight‐corrected tidal volume (VThf/kg), oscillatory frequency, mean airway pressure, FiO_2_, DCO_2_, and variance decomposition of oscillatory parameters.

The clinical outcome of interest was the composite of death before discharge or moderate‐to‐severe bronchopulmonary dysplasia at 36 weeks of postmenstrual age.

### 2.8 Statistical Analysis

Continuous variables are presented as median and interquartile range and categorical variables as counts. Analyses used available‐case data without imputation. The numbers of infants and hourly windows contributing to each predefined epoch (0–6, 6–12, 12–24, 24–48, and 48–72 h) are reported explicitly.

Hourly oscillatory amplitude variability was quantified using standard deviation, coefficient of variation (CV), and root mean square of successive differences (RMSSD). CV and RMSSD were log‐transformed before modelling because of right‐skewed distributions. Associations with ventilator variables were assessed using linear mixed‐effects models with infant‐specific random intercepts. Time‐varying covariates were partitioned into within‐infant deviations and between‐infant means, with time from HFOV initiation modelled as a log‐transformed continuous variable. Time‐by‐within‐infant interaction terms were included to assess whether associations changed over time. Variance components were used to calculate intraclass correlation coefficients and quantify within‐ and between‐infant variability.

Residual serial autocorrelation was assessed, and robustness of the longitudinal findings was evaluated using a first‐order autoregressive [AR(1)] within‐infant correlation structure. Model specification, covariance assessment, diagnostics, and sensitivity analyses are provided in the Supplementary Statistical Methods.

For infant‐level discrimination analyses, early amplitude CV was calculated across all valid minute‐level amplitude observations during the 0–24 h interval. Discrimination for death before discharge or moderate‐to‐severe bronchopulmonary dysplasia was assessed using receiver operating characteristic analysis for early CV alone and in combination with mean airway pressure, FiO_2_, oxygenation index, oscillatory frequency, and weight‐normalised DCO_2_. AUCs with 95% confidence intervals were reported, with sensitivity and specificity at the optimal Youden threshold. Given the modest cohort size and small number of infants without the composite outcome, these analyses were considered exploratory and hypothesis‐generating and were not intended to derive or validate a clinical prediction model.

Statistical significance was defined as a two‐sided *p* < 0.05. Original analyses were conducted using Python version 3.10 and IBM SPSS Statistics version 31.0; additional longitudinal sensitivity and diagnostic analyses were performed using Python version 3.13.5. Further software and implementation details are provided in the Supplementary Statistical Methods.

### 2.9 Ethics Statement and Approval

This study was approved by the Institutional Review Board of King Abdullah International Medical Research Centre (KAIMRC), Riyadh, Saudi Arabia (IRB number 00000157825). The requirement for informed consent was waived due to the retrospective nature of the study and the use of de‐identified data. The study was conducted in accordance with the principles of the Declaration of Helsinki.

## 3 Results

### 3.1 Cohort Characteristics

The cohort comprised 60 infants with a median gestational age of 26.0 (25.0–28.0) weeks and birth weight of 860 (700–1000) g (Table 1). The composite outcome of death before discharge or moderate to severe BPD occurred in 47/60 (78.3%) infants. Longitudinal analyses were restricted to the prespecified first 72 h after NICU admission/initiation of HFOV‐VG.

**Table 1:** Cohort characteristics, clinical outcomes and monitoring structure.

Characteristic	Value
Cohort characteristics	
Infants, *n*	60
Gestational age, weeks	26.0 (25.0–28.0)
Birth weight, g	860 (700–1000)
Male sex, *n* (%)	26/60 (43.3)
Antenatal corticosteroid exposure, *n* (%)	46/60 (76.7)
Surfactant exposure, *n* (%)	60/60 (100)
Clinical outcomes	
Death before discharge, *n/N* (%)	6/60 (10)
BPD at 36 weeks, *n* (%)	42/60 (70.0)
Composite death or moderate‐to‐severe BPD, *n* (%)	47/60 (78.3)
Pneumothorax, *n* (%)	4/60 (6.7)
Postnatal corticosteroid exposure, *n* (%)	40/60 (66.7)
ROP, *n* (%)	14/60 (23.3)
IVH, *n* (%)	15/60 (25.0)
Sepsis, *n* (%)	8/60 (13.3)
NEC, *n* (%)	7/60 (11.7)
Monitoring and ventilatory characteristics	
Prespecified longitudinal analytical window, h	0–72
Amplitude, mbar	17.00 (14.75–20.00)
VThf/kg, mL/kg	1.73 (1.50–1.94)
Frequency, Hz	15.00 (15.00–15.00)
MAP, mbar	10.00 (10.00–12.00)
FiO_2_, %	30.0 (25.0–35.0)

*Note:* Values are presented as *n*, *n/N* (%), or median (interquartile range). Mortality status was unavailable for one infant and is therefore reported using the available denominator (*n* = 59). The composite outcome was death before discharge or moderate‐to‐severe BPD at 36 weeks' postmenstrual age.

Abbreviations: BPD, bronchopulmonary dysplasia; FiO2, fraction of inspired oxygen; IVH, intraventricular haemorrhage; MAP, mean airway pressure; NEC, necrotising enterocolitis; ROP, retinopathy of prematurity; VThf/kg, weight‐corrected oscillatory tidal volume.

### 3.2 Temporal Changes in Oscillatory Variability and Ventilator Parameters

Oscillatory amplitude variability decreased across successive epochs (Table 2). CV declined from 0.122 (0.072–0.160) at 0 to 6 h to 0.063 (0.035–0.106) at 48 to 72 h, while RMSSD decreased from 2.10 (1.22–2.69) to 1.13 (0.64–1.94) mbar. Over the same period, VThf/kg increased, while MAP and FiO_2_ generally decreased. The number of contributing infants varied across epochs because electronic recording did not commence uniformly from admission.

**Table 2:** Temporal changes in oscillatory amplitude variability, ventilator parameters, and carbon dioxide transport during the first 72 h of HFOV with volume guarantee.

Panel A. Oscillatory amplitude and variability						
Time epoch	Hourly windows (*n*)	Infants (*n*)	Amplitude (mbar)	SD (mbar)	CV	RMSSD (mbar)
0–6 h	63	14	17.42 (15.53–20.94)	2.07 (1.33–2.88)	0.122 (0.072–0.160)	2.10 (1.22–2.69)
6–12 h	134	33	16.29 (13.71–20.05)	1.76 (0.88–2.77)	0.101 (0.054–0.176)	1.46 (0.82–2.63)
12–24 h	614	58	17.09 (13.39–19.92)	1.24 (0.66–2.17)	0.077 (0.042–0.140)	1.20 (0.68–2.13)
24–48 h	1239	58	17.07 (13.99–19.99)	0.97 (0.50–1.82)	0.062 (0.034–0.106)	0.96 (0.54–1.74)
48–72 h	989	48	18.40 (15.48–21.42)	1.12 (0.58–2.02)	0.063 (0.035–0.106)	1.13 (0.64–1.94)

Panel B. Ventilator parameters and carbon dioxide transport					
Time epoch	VThf/kg (mL/kg)	Frequency (Hz)	MAP (mbar)	FiO_2_ (%)	DCO_2_ (mL^2^/s/kg)
0–6 h	1.58 (1.47–1.70)	15.00 (15.00–15.41)	11.87 (10.00–13.00)	57.5 (33.8–80.0)	26.1 (20.5–46.0)
6–12 h	1.62 (1.41–1.81)	15.00 (15.00–15.00)	11.00 (10.00–12.00)	47.1 (29.4–70.0)	24.3 (16.4–38.1)
12–24 h	1.68 (1.51–1.88)	15.00 (15.00–15.00)	11.00 (10.00–12.00)	35.0 (26.0–53.4)	29.0 (18.2–48.3)
24–48 h	1.69 (1.50–1.90)	15.00 (15.00–15.00)	10.98 (9.35–12.00)	28.9 (25.0–35.2)	32.7 (18.1–48.7)
48–72 h	1.72 (1.51–1.99)	15.00 (15.00–15.00)	10.00 (9.00–12.00)	30.5 (25.1–39.0)	29.1 (19.0–41.5)

*Note:* Values are presented as median (interquartile range). CV was calculated as SD divided by mean amplitude; RMSSD represents the root mean square of successive differences in amplitude.

Abbreviations: CV, coefficient of variation; DCO2, diffusion coefficient of carbon dioxide; FiO2, fraction of inspired oxygen; MAP, mean airway pressure; RMSSD, root mean square of successive differences; SD, standard deviation; VThf/kg, weight‐corrected tidal volume.

### 3.3 Variance Structure of Oscillatory Parameters

Within‐infant variation accounted for 75.4% of total variance in amplitude CV and 71.8% in RMSSD (Table 3). In contrast, between‐infant variation accounted for the majority of variance in mean amplitude, VThf/kg, and oscillatory frequency, with frequency having the highest ICC (0.761).

**Table 3:** Variance decomposition of oscillatory parameters during HFOV with volume guarantee.

Variable	Between‐infant variance	Within‐infant variance	Total variance	ICC	Within‐infant contribution (%)	Dominant variance structure
Amplitude CV	0.001	0.003	0.004	0.246	75.4	Predominantly within‐infant temporal variability
Amplitude RMSSD (mbar)	0.310	0.789	1.099	0.282	71.8	Predominantly within‐infant temporal variability
Amplitude (mean, mbar)	14.656	10.743	25.399	0.577	42.3	Predominantly between‐infant variability
VThf/kg (mL/kg)	0.098	0.039	0.137	0.718	28.2	Predominantly between‐infant variability
Frequency (Hz)	1.345	0.422	1.767	0.761	23.9	Predominantly between‐infant variability

*Note:* Values are presented as variance components derived from mixed‐effects models.

Abbreviations: CV, coefficient of variation; ICC, proportion of total variance attributable to between‐infant differences; RMSSD, root mean square of successive differences; VThf/kg, weight‐corrected tidal volume.

### 3.4 Multivariable Associations With Oscillatory Variability

Within‐infant increases in oscillatory frequency were associated with higher variability, whereas increases in MAP and time from HFOV initiation were associated with lower variability (Table 4). Each 1 Hz increase in frequency was associated with 66.9% higher CV (95% CI 8.1% to 157.5%; *p* = 0.021) and 80.8% higher RMSSD (95% CI 14.5% to 185.8%; *p* = 0.011). Each 1 mbar increase in MAP was associated with 6.8% lower CV (*p* = 0.021) and 7.5% lower RMSSD (*p* = 0.019). VThf/kg was not significantly associated with either measure. The frequency by time interaction was significant for RMSSD (*p* = 0.037) but not for CV (*p* = 0.053). AR(1) sensitivity analyses and model diagnostics are presented in the Supplementary Material.

**Table 4:** Multivariate associations with oscillatory amplitude variability during HFOV with volume guarantee.

Covariate	Amplitude CV % difference (95% CI)	*p* value	Amplitude RMSSD % difference (95% CI)	*p* value
Within‐infant temporal effects				
Frequency deviation (per 1 Hz increase)	66.9% (8.1% to 157.5%)	0.021	80.8% (14.5% to 185.8%)	0.011
MAP deviation (per 1 mbar increase)	−6.8% (−12.2% to −1.0%)	0.021	−7.5% (−13.2% to −1.3%)	0.019
VThf/kg deviation (per 0.5 mL/kg increase)	−61.7% (−91.2% to 67.4%)	0.202	−68.0% (−92.2% to 30.3%)	0.112
Time‐dependent effects				
Time from HFOV initiation (log‐transformed)	−20.0% (−28.5% to −10.5%)	< 0.001	−15.5% (−24.9% to −5.0%)	0.005
Frequency deviation × time	−13.8% (−25.8% to 0.2%)	0.053	−15.6% (−28.0% to −1.0%)	0.037
Between‐infant effects				
Mean MAP	−8.1% (−14.8% to −1.0%)	0.027	−4.4% (−12.6% to 4.7%)	0.335

*Note:* Both mixed‐effects models included 2,963 infant‐hour observations from 58 infants with complete data for the variables included in the models. CV and RMSSD were log‐transformed; coefficients and 95% CIs were exponentiated and expressed as percentage differences [100 × (exp(β) − 1)]. Estimates for frequency and MAP represent within‐infant changes per 1 Hz and 1 mbar, respectively, and those for VThf/kg represent changes per 0.5 mL/kg. Time and interaction effects were modelled on the log‐time scale.

Abbreviations: CV, coefficient of variation; MAP, mean airway pressure; RMSSD, root mean square of successive differences; VThf/kg, weight‐corrected oscillatory tidal volume.

### 3.5 Predictive Performance of Early Oscillatory Amplitude Variability

Early amplitude CV had an AUC of 0.72 (95% CI 0.55–0.86) for death or moderate to severe BPD (Table 5). The highest AUC was observed for the combination of early CV, oscillatory frequency, and DCO_2_/kg (AUC 0.82, 95% CI 0.66–0.95).

**Table 5:** Predictive performance of early oscillatory amplitude variability for adverse respiratory outcome.

Model	*n*	Events	AUC (95% CI)	Youden threshold	Sensitivity, % (95% CI)	Specificity, % (95% CI)
Early amplitude CV alone	60	47	0.72 (0.55–0.86)	0.127	87.2 (74.8–94.0)	61.5 (35.5–82.3)
Early CV + MAP + FiO_2_	59	46	0.73 (0.55–0.88)	0.751	78.3 (64.4–87.7)	69.2 (42.4–87.3)
Early CV + OI	57	44	0.73 (0.56–0.87)	0.663	88.6 (76.0–95.0)	61.5 (35.5–82.3)
Early CV + frequency	59	46	0.72 (0.57–0.87)	0.691	87.0 (74.3–93.9)	61.5 (35.5–82.3)
Early CV + DCO_2_/kg	60	47	0.76 (0.60–0.89)	0.735	85.1 (72.3–92.6)	61.5 (35.5–82.3)
Early CV + frequency + DCO_2_/kg	59	46	0.82 (0.66–0.95)	0.568	93.5 (82.5–97.8)	69.2 (42.4–87.3)

*Note:* The outcome was death before discharge or moderate‐to‐severe BPD at 36 weeks of postmenstrual age. Thresholds were selected using the Youden index; for multivariable models, thresholds represent model‐predicted probabilities. Sensitivity and specificity are presented with Wilson 95% CIs.

Abbreviations: AUC, area under the receiver operating characteristic curve; CV, coefficient of variation; DCO2/kg, weight‐normalised diffusion coefficient of carbon dioxide; FiO2, fraction of inspired oxygen; MAP, mean airway pressure; OI, oxygenation index.

## 4 Discussion

In this time‐resolved cohort study, oscillatory amplitude variability during high‐frequency oscillatory ventilation with volume guarantee (HFOV‐VG) showed a distinct temporal pattern, with greater variability early after HFOV initiation followed by progressive stabilisation. Variance decomposition demonstrated that amplitude variability arose predominantly within infants over time, whereas mean oscillatory amplitude, tidal volume delivery, and oscillatory frequency showed greater between‐infant variation. Multivariate modelling identified associations of variability with dynamic changes in oscillatory frequency, mean airway pressure, and time from HFOV initiation, consistent with amplitude variability reflecting evolving ventilator‐lung interaction during HFOV‐VG. Early amplitude variability also showed discriminatory ability for death or moderate‐to‐severe bronchopulmonary dysplasia, with higher apparent discrimination when combined with other indices of oscillatory ventilation. Given the exploratory nature of the outcome analyses, these findings support amplitude variability as a potential physiological marker rather than an established clinical predictor.

Multivariate modelling demonstrated that within‐infant increases in oscillatory frequency were associated with higher variability, whereas increases in mean airway pressure and time from HFOV initiation were associated with lower variability. These associations are physiologically plausible and may reflect frequency‐dependent changes in respiratory system impedance and oscillatory pressure transmission during HFOV. Ventilation during oscillation is influenced by the interaction between resistance, compliance, and inertance, which together determine respiratory impedance at a given frequency [13, 14, 15]. Higher oscillatory frequencies are associated with reduced transmission of oscillatory pressure to peripheral lung regions and smaller delivered oscillatory volumes [1, 13]. In this context, the greater variability observed during the early phase of HFOV‐VG may reflect evolving lung recruitment, heterogeneous compliance, and changing respiratory mechanics within the immature lung, including relatively rapid changes following interventions such as surfactant administration and lung recruitment manoeuvres. The progressive reduction in variability over time may similarly be consistent with increasing mechanical stability and more homogeneous ventilation as respiratory adaptation progresses [14, 16, 17, 18].

These findings are consistent with established physiological principles governing gas exchange during HFOV, in which oscillatory pressure generated at the airway opening undergoes progressive damping as it is transmitted through the respiratory system [1, 9, 19, 20]. Oscillatory pressure transmission is influenced by lung compliance, airway resistance, regional heterogeneity, and end‐expiratory lung volume [1, 2, 11, 19, 21]. Importantly, these findings were observed during HFOV‐VG, in which delivered tidal volume is actively stabilised through automatic adjustment of oscillatory pressure amplitude in response to changes in respiratory mechanics [7, 22, 23, 24]. Recent systematic evidence similarly suggests that volume‐targeted HFOV reduces variability in delivered oscillatory tidal volume, although evidence regarding amplitude behaviour remains limited [25]. The persistence of oscillatory amplitude variability despite relatively stable VThf therefore suggests that the amplitude adjustments required to maintain targeted volume may provide information about evolving ventilator‐lung interaction beyond tidal volume delivery alone. This relationship provides a physiological basis for amplitude variability as a potential marker of changing respiratory system behaviour during HFOV‐VG [26].

Further physiological context is provided by studies demonstrating that lung recruitment during HFOV is a dynamic and time‐dependent process rather than an immediate response to ventilator adjustment. Tingay et al. demonstrated that following changes in mean airway pressure during HFOV, attainment of stable lung volume frequently required prolonged periods and varied according to the preceding lung volume state, with slower stabilisation observed in poorly recruited lungs [27]. This time‐dependent recruitment response provides a plausible physiological explanation for the greater amplitude variability observed early after HFOV initiation. The subsequent decline in variability may similarly reflect progressive stabilisation of lung volume and more homogeneous mechanical behaviour as recruitment progresses [28].

Beyond its physiological interpretation, amplitude variability may also provide clinically relevant information during the early phase of HFOV‐VG. Early oscillatory amplitude variability demonstrated discriminatory ability for death or moderate‐to‐severe bronchopulmonary dysplasia, with higher apparent discrimination when combined with indices of oscillatory ventilation. From a clinical perspective, this raises the possibility that variability‐based measures may assist in identifying infants with evolving respiratory instability not fully reflected by conventional ventilator‐derived parameters. At present, however, these measures should be considered physiological monitoring markers rather than parameters for directing ventilator adjustments, and their clinical utility and actionable thresholds require prospective validation.

This study has several strengths. The use of high‐resolution, minute‐level ventilator data during the first 72 h of HFOV‐VG enabled detailed characterisation of oscillatory amplitude variability over time beyond that achievable with intermittent sampling. The analytical framework further incorporated mixed‐effects modelling with separation of within‐infant and between‐infant effects, allowing dynamic temporal associations to be distinguished from between‐infant differences and enabling variance decomposition of oscillatory parameters.

Several limitations should be considered. The retrospective design may have introduced confounding related to clinical management decisions and illness severity. Amplitude variability may also have been influenced by unmeasured clinical factors, including patient handling, respiratory distress, adequacy of ventilatory support, and sedation, which could not be reliably characterised from the retrospective dataset. In particular, we could not determine whether greater early variability reflected inadequate or evolving ventilatory support, and the subsequent decline in variability may partly reflect progressive optimisation of ventilator settings rather than changes in respiratory mechanics alone. Although all infants commenced HFOV‐VG on NICU admission, electronically captured ventilator data were not uniformly available from the earliest hours in every infant. Recording availability therefore varied across epochs and may not have been random, particularly during the 0–6 and 6–12 h epochs. Direct measurements of lung volume, regional ventilation distribution, and respiratory mechanics were not available; oscillatory amplitude variability therefore represents an indirect measure of respiratory system behaviour, limiting mechanistic interpretation. Endotracheal tube size and changes in tube size during HFOV‐VG were not consistently available and could not be incorporated into the analyses; because ETT characteristics may influence oscillatory pressure transmission, residual confounding related to airway instrumentation cannot be excluded. The study was conducted in a single centre using a single ventilator platform. VG control algorithms and ventilator responses may differ between ventilator platforms, which may limit generalisability to other devices and settings, although the use of a single platform reduced device‐related heterogeneity within the cohort. The clinical discrimination analyses were exploratory and based on a modest cohort of 60 infants, including only 13 without the composite outcome. Multivariable estimates may therefore be susceptible to model instability, overfitting, and optimistic apparent discrimination and require validation in a larger independent cohort.

## 5 Conclusion

Oscillatory amplitude variability during HFOV‐VG in preterm infants demonstrated structured temporal behaviour, with variability arising predominantly within infants over time. Variability was greatest early after HFOV initiation and declined progressively, consistent with evolving ventilator‐lung interaction and respiratory mechanics. Early variability showed discriminatory ability for death or moderate‐to‐severe bronchopulmonary dysplasia, with higher apparent discrimination when combined with indices of oscillatory ventilation. These findings support further investigation of amplitude variability as a physiological monitoring marker, but its clinical utility and actionable thresholds require prospective validation in larger independent cohorts.

## Author Contributions

**Kamal Ali:** conceptualisation, investigation, writing – original draft, methodology, validation, visualisation, writing – review and editing, software, formal analysis, project administration, supervision, resources. **Mesaed Alsenani:** investigation, methodology, writing – review and editing, data curation. **Saad Alshareedah:** investigation, writing – review and editing, methodology, data curation. **Mohamed Sufyani:** investigation, methodology, writing – review and editing, data curation. **Saleh Algarni:** investigation, methodology, visualisation, writing – review and editing, data curation. **Piero Alberti:** investigation, methodology, visualisation, writing – review and editing, data curation. **Abdulaziz Homedi:** investigation, methodology, visualisation, writing – review and editing, data curation. **Saif Alsaif:** investigation, methodology, writing – review and editing. **Ibrahim Ali:** investigation, methodology, writing – review and editing. **Theodore Dassios:** conceptualisation, investigation, writing – review and editing, methodology, formal analysis, supervision, resources. **Anne Greenough:** conceptualisation, investigation, methodology, validation, visualisation, writing – review and editing, formal analysis, project administration, supervision, resources.

## Funding

The authors have nothing to report.

## Conflicts of Interest

The authors declare no conflicts of interest.

## Supporting information


Supporting File

## Data Availability

The datasets generated and/or analysed during the current study are not publicly available due to institutional data protection policies but are available from the corresponding author on reasonable request and with permission from King Abdulaziz Medical City.
