# Supplementary material for: Temporal Dynamics and Clinical Relevance of Oscillatory Amplitude Variability During High‐Frequency Oscillatory Ventilation With Volume Guarantee in Preterm Infants
Source: Pediatr Pulmonol. 2026 Oct 4;61(10):e71859. doi: 10.1002/ppul.71859 (PMC13634310; doi:10.1002/ppul.71859)
Supplement: Supplementary file 1 [file PPUL-61-0-s001.docx]

**Oscillatory amplitude variability during high-frequency oscillatory ventilation with volume guarantee reflects early risk of adverse outcome in preterm infants**

**Supplementary Statistical Methods**

Hourly amplitude coefficient of variation (CV) and root mean square of successive differences (RMSSD) were analysed as longitudinal outcomes. Both outcomes were natural-log transformed before regression modelling because of right-skewed distributions. Time-varying ventilator covariates were decomposed into within-infant and between-infant components using person-mean centring. For each covariate, the within-infant component represented the deviation of the hourly value from that infant’s mean across the analysed period, whereas the infant-specific mean represented the between-infant component. Time from HFOV-VG initiation was entered as a log-transformed continuous variable, and time-by-within-infant interaction terms were included to assess whether associations changed over time.

The primary longitudinal analyses used linear mixed-effects models with an infant-specific random intercept to account for clustering of repeated hourly observations within infants and to estimate within-infant and between-infant associations separately. Fixed effects included within-infant deviations in ventilator variables, between-infant mean ventilator variables, log-transformed time from HFOV-VG initiation, and interactions between time and within-infant covariates. Separate models were fitted for log(CV) and log(RMSSD).

The random-intercept specification accounts for correlation attributable to repeated observations from the same infant and permits estimation of between-infant heterogeneity but does not impose a time-ordered residual covariance structure. Residual serial dependence was therefore assessed within infants, with particular attention to lag-1 residual autocorrelation. Robustness of the longitudinal findings to residual serial correlation was evaluated using a first-order autoregressive [AR(1)] within-infant correlation structure with the same fixed-effect specification. The AR(1) sensitivity analysis allowed correlation between repeated observations to decrease with increasing temporal separation.

Model diagnostics included assessment of residual serial correlation and multicollinearity among fixed-effect covariates. Variance inflation factors (VIFs) were calculated for the fixed-effect design matrix. As no minimum number of minute-level observations per hourly window had been prespecified, sensitivity analyses requiring at least 30 valid amplitude observations per hour were performed to assess the robustness of the hourly variability estimates and longitudinal findings.

**Supplementary Table S1. Serial-correlation and collinearity diagnostics**

| **Diagnostic** | **log(CV)** | **log(RMSSD)** | **Interpretation** |
| --- | --- | --- | --- |
| Infants, n | 60 | 60 | Independent participants |
| Median within-infant lag-1 residual autocorrelation | 0.29 | 0.44 | Residual serial dependence was present after accounting for infant-level clustering |
| Estimated AR(1) correlation parameter | 0.53 | 0.62 | Moderate positive correlation between temporally adjacent within-infant observations |
| Maximum VIF | 1.18 | 1.18 | No evidence of important multicollinearity among fixed-effect covariates |

*CV, coefficient of variation; RMSSD, root mean square of successive differences; AR(1), first-order autoregressive correlation structure; VIF, variance inflation factor. Serial-correlation diagnostics were derived from the hourly sensitivity analysis. Values are reported to two decimal places.*

In the serial-correlation sensitivity analysis, median within-infant lag-1 residual autocorrelation was 0.29 for log(CV) and 0.44 for log(RMSSD), with corresponding AR(1) correlation parameters of 0.53 and 0.62. These findings demonstrate residual temporal dependence between successive hourly observations beyond that represented by the infant-specific random intercept alone. Multicollinearity was minimal, with a maximum VIF of 1.18.

The random-intercept mixed-effects framework permitted decomposition of within-infant and between-infant effects but did not fully account for residual temporal dependence between successive hourly observations. The AR(1) sensitivity analysis was therefore used to assess the influence of this residual serial correlation on longitudinal inference.

**Software**

The supplementary longitudinal diagnostic and sensitivity analyses were performed using Python version 3.13.5 with pandas version 2.2.3, NumPy version 2.3.5, and statsmodels version 0.14.6. Original analyses were conducted using Python version 3.10 and IBM SPSS Statistics for Windows, version 31.0.
